# Cerebral magnetic resonance spectroscopy – insights into preterm brain injury

**DOI:** 10.1038/s41372-024-02172-2

**Published:** 2024-11-28

**Authors:** Magdalena Zasada, Paulina Karcz, Marta Olszewska, Aleksandra Kowalik, Wojciech Zasada, Izabela Herman-Sucharska, Przemko Kwinta

**Affiliations:** 1 Department of Pediatrics, Jagiellonian University Medical College, Krakow, Poland; 2 Department of Electroradiology, Jagiellonian University Medical College, Faculty of Health Sciences, Krakow, Poland; 3 Clinical Department of Cardiology and Cardiovascular Interventions, University Hospital, Krakow, Poland; 4 Department of Radiology, Jagiellonian University Medical College, Faculty of Health Sciences, Krakow, Poland

**Keywords:** Medical research, Neuroscience

## Abstract

**Objective:**

Magnetic resonance spectroscopy (^1^H-MRS) may provide clinically relevant data regarding metabolic processes that govern the course of preterm brain injury.

**Study design:**

46 very preterm infants (VP) were evaluated by magnetic resonance imaging and ^1^H-MRS at term-equivalent age. Brain injury was assessed according to the Kidokoro scale. Moreover, 17 term-born infants with hypoxic-ischemic encephalopathy (HIE) were scanned. The metabolic profile of the central nervous system was obtained from the bilateral thalamus.

**Result:**

The Lipids/Creatine, Choline/Creatine, N-acetyl aspartate/Choline, Lactate/N-acetyl aspartate, and Lactate/Creatine ratios differed between VP infants with moderate+severe brain damage and those without brain injury. Moreover, VP infants with moderate+severe brain damage had higher Lactate/ N-acetyl aspartate and Lactate/Creatine ratios than HIE group.

**Conclusion:**

There were significant differences in the cerebral metabolite profile at TEA between VP infants with and without brain injury. The ^1^H-MRS profile of VP infants with moderate+severe brain damage may reflect profound chronic metabolic alterations.

## Introduction

Neonatal brain injury is a significant cause of mortality and morbidity, often with life-long consequences. In premature babies, the most common brain injuries are germinal matrix hemorrhage-intraventricular hemorrhage (GMH-IVH), periventricular leukomalacia (PVL) and diffuse white matter injury (dWMI). In neonates born at term, hypoxic-ischemic encephalopathy (HIE) follows an episode of hypoxia-ischemia. A better understanding of the processes that govern the course of preterm brain injury may help in identifying pathomechanisms that may be targeted by new therapeutic interventions.

In premature infants, hypoxia-ischemia, GMH-IVH and WMI are associated with thalamic injury, which may have further neurodevelopmental consequences. Interestingly, there is an evidence of uneven susceptibility of both thalami to prematurity associated damage [1].

Magnetic resonance imaging (MRI) techniques provide valuable anatomical information about the newborn brain. For very preterm children, a detailed cerebral morphology assessment scoring system has been developed [2]. Proton magnetic resonance spectroscopy (^1^H-MRS) is used to determine the biochemical composition of living tissues and may provide clinically relevant data regarding differences in metabolic processes occurring in the brains of infants with and without neonatal brain injury [3–5].

As ^1^H-MRS data obtained from children with HIE are much better described and explored [3, 6], a comparison of these data with the results of cerebral spectroscopy in preterm infants may increase our understanding of the pathophysiology of preterm brain injury. To date, studies that have used the same methodology to perform ^1^H-MRS in premature infants and full-term asphyxiated infants are limited, which makes comparisons difficult, as research carried out using different devices and diverse parameters may complicate performing reliable assessment of the obtained results.

The aim of this study was to enhance our understanding of brain injury patterns in preterm-born infants. For this purpose, we analyzed and compared the ratios of selected brain metabolites assessed in ^1^H-MRS in bilateral thalamus in groups of very preterm (VP) infants with and without neonatal brain injury. Additionally, we compared them with ratios obtained from term-born infants with HIE.

## Materials and methods

### Enrolled patients

This single-center, prospective study was conducted in the Neonatal Intensive Care Unit (NICU), Institute of Pediatrics, Jagiellonian University Medical College, Cracow, Poland. Between April 2021 and July 2023, two cohorts of infants were consecutively and simultaneously enrolled:

1. VP newborns with a gestational age (GA) of 22^0/7^–31^6/7^ weeks admitted to the NICU within 0–24 h of life.
2. Newborns with a GA ≥ 35^0/7^ weeks with moderate to severe HIE (according to the Sarnat & Sarnat scale) who qualified for therapeutic hypothermia [7] and were admitted to the NICU within 0–6 h of life.

**Fig. 1:**
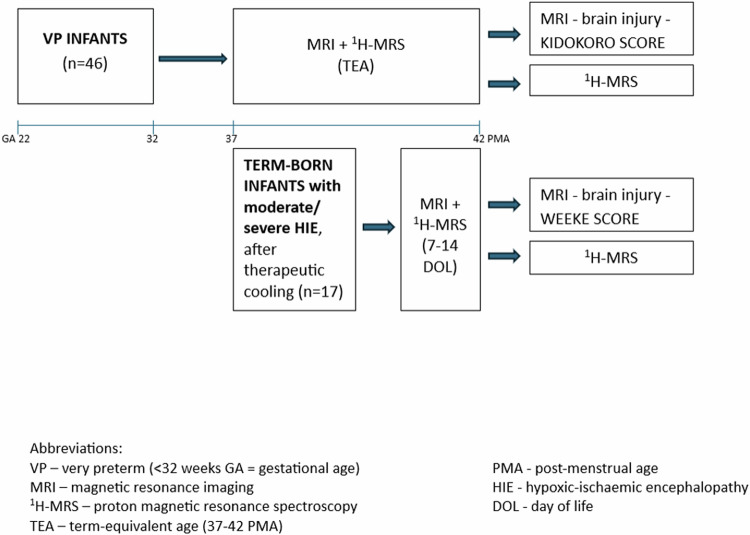
Study design.

The exclusion criteria for both cohorts were as follows: (1) major congenital anomalies of the heart/kidney or any structural abnormalities on cranial ultrasound (cUS) upon admission, (2) multiple pregnancies, and (3) clinical suspicion of metabolic/genetic disorders (Fig. 1).

### Monitoring during hospitalization

All study participants underwent clinical monitoring for symptoms of neonatal brain injury, which is the standard of care. IVH occurrence and grade were assessed via cUS. PVL and dWMI presence and severity were evaluated according to cUS and MRI findings. CUSs were performed as the standard of care according to the management guidelines by attending physicians certified by the Polish Ultrasound Society.

### Data collection

Patient data, involving perinatal history, hospitalization course and the incidence of prematurity complications with special regard to the occurrence of neonatal brain injury, were prospectively collected.

### MRI and ^1^H-MRS acquisition

The patients were subjected to MRI and ^1^H-MRS:

1. VP infants - at term-equivalent age (TEA) (37–42 PMA).
2. Neonates with HIE - between 7 and 14 days of life (DOL).

The details of the performed examinations can be found in the Supplementary Materials.

### MRI scoring system

MRI images of the VP infants were scored for the presence of brain damage, using the Kidokoro scoring system [2]. In addition to the assessment strictly according to Kidokoro, each symmetrical part of the brain was assessed separately on the left side and separately on the right side, and the results of this evaluation were also recorded.

MRI images of the HIE infants were also scored for the presence of brain injury, using the Weeke scoring system [8].

Analyses were performed by four pediatricians and neonatologists (PK, MZ, AK, MO) who were previously trained by a neonatal neuroradiologist with >30 years of experience (IHS), using the program RadiAnt DICOM Viewer. Readers were blinded to long-term outcomes and clinical data except for gestational age and post-menstrual age (PMA). Two reviewers assessed every examination. Any disagreements in grading were resolved by consensus with neonatal neuroradiologist.

### Statistical analysis

Categorical variables were presented as numbers and percentages. Because the distribution of the analyzed continuous variables, verified by the Shapiro‒Wilk test, differed in each case from the normal distribution, continuous variables were presented as medians with the first and third quartiles (Q1‒Q3). Pearson’s chi-square test was used to evaluate categorical variables, while the Wilcoxon test was used for continuous variables. For multiple comparisons of continuous variables, the Wilcoxon signed-rank test was used, and for categorical variables, Pearson’s chi-square test with Bonferroni correction was used. Studied groups were compared directly (crude data) and after standardization for PMA (adjusted data). A two-sided *p* value less than 0.05 denoted statistical significance. JMP®, Version 17.1.0 (JMP Statistical Discovery) was used for all the statistical analyses.

## Results

A total of 63 patients, including 46 VP neonates and 17 full-term neonates with HIE subjected to therapeutic hypothermia, were included. VP neonates were further divided into three subgroups according to the Kidokoro grading system depending on the severity of brain damage: no damage (global brain abnormality score (GBAS) indicating normality, 23 patients), mild damage (GBAS indicating mild abnormalities, 15 patients) and moderate/severe damage (GBAS indicating moderate/severe abnormalities, 8 patients). Infants from the HIE group were assessed according to Weeke score, received from 0 to 11 points, median was 2 points (Q1–Q3: 1–4.5 points). The demographic data of the patients are presented in Table 1.

**Table 1:** Clinical characteristics of patients.

	VP *n* = 46			*p*	HIE *n* = 17	p VP vs. HIE
	GBAS normal *n* = 23	GBAS mild *n* = 15	GBAS moderate+severe *n* = 8			
Gestational age, weeks; median (Q1–Q3)	30 (28–30)	29 (27–30)	28 (27.25–29)	0.1145	39 (38–40)	<0.0001
Birth weight, grams; median (Q1–Q3)	1350 (1150–1560)	1400 (950–1490)	1250 (1020–1467.5)	0.6630	3350 (3155–3650)	<0.0001
Male gender; *n* (% of group)	11 (47.83)	10 (66.67)	5 (62.50)	0.5343	10 (58.82)	0.8698
Postconceptional age when performing MRI + ^1^H-MRS, weeks; median (Q1–Q3)	39 (38–39)	37 (37–40)	39 (38–40)	0.3670	40 (39.5–41)	<0.0001

*VP* very preterm infants, *HIE* infants with hypoxic-ischemic encephalopathy, *GBAS* global brain abnormality score.

### Comparison of ^1^H-MRS metabolite ratios between left and right thalami

According to the ^1^H-MRS results, in VP infants, two different metabolite ratios were found between the left and right thalami: the Cho/Cr ratio, which was significantly greater in the left thalamus (*p* = 0.0193), and the NAA/Cho ratio, which was significantly lower in the left thalamus (*p* = 0.0181). In the HIE group, no statistically significant differences were found between the right and left thalami (Table 2).

**Table 2:** Comparison of metabolite ratios from the left and right thalami both in VP infants and in full-term infants with HIE.

Metabolite ratio		HIE		*p*	VP		*p*
		LT	RT		LT	RT	
Lac/NAA	Median	0.1677	0.1418	0.3987	0.1961	0.1694	0.6225
	Q1	0.1023	0.1010		0.1192	0.1171	
	Q3	0.2408	0.1865		0.3024	0.2555	
Lac/Cr	Median	0.2640	0.1773	0.1965	0.2335	0.2162	0.5881
	Q1	0.1427	0.1247		0.1472	0.1491	
	Q3	0.3636	0.2773		0.3795	0.3296	
Lip/Cr	Median	0.2741	0.2370	0.7962	0.2831	0.2354	0.6876
	Q1	0.1251	0.1700		0.2049	0.1797	
	Q3	0.3350	0.2850		0.3354	0.3809	
Cho/Cr	Median	1.0790	1.2783	0.3614	1.2246	1.0996	0.0193
	Q1	0.9745	1.0384		1.1101	0.9393	
	Q3	1.3138	1.4410		1.4374	1.2888	
NAA/Cr	Median	1.3868	1.3797	0.9588	1.2228	1.1655	0.5712
	Q1	1.2505	1.1477		1.0535	1.0103	
	Q3	1.5894	1.6967		1.4364	1.3643	
NAA/Cho	Median	1.2248	1.1618	0.5467	0.9388	1.1296	0.0181
	Q1	1.0334	0.9426		0.8355	0.9519	
	Q3	1.3836	1.4009		1.0930	1.2545	

*LT* left thalamus, *RT* right thalamus.

Comparison of neonatal brain injury on the right and left sides of the brain in the group of premature newborns, visible by cranial ultrasound and/or brain MRI, revealed no significant differences between the two sides of the brain (Table 3).

**Table 3:** Comparison of preterm brain injury on the right and left sides of the brain in the group of VP infants, detected by cUS and/or brain MRI.

	Brain side		*p*
	Left *n* = 46	Right *n* = 46	
**Cranial Ultrasound**			
Intraventricular hemorrhage grade (according to Papille); *n* (%)			0.5947
No	30 (65.22)	29 (63.04)	
I	2 (4.35)	4 (8.70)	
II	5 (10.87)	3 (6.52)	
III	6 (13.04)	9 (19.57)	
IV	3 (6.52)	1 (2.17)	
Periventricular leukomalacia grade (according to de Vries); *n* (%)			0.9950
No	26 (56.52)	28 (60.87)	
I	16 (34.78)	14 (30.43)	
II	2 (4.35)	2 (4.35)	
III	1 (2.17)	1 (2.17)	
IV	1 (2.17)	1 (2.17)	
Higher IVH grade; *n* (%)	4 (8.70)	1 (2.17)	0.1355
**Magnetic resonance imaging (according to Kidokoro score)**			
White matter cystic lesions; *n* (%)			0.5134
Extensive	4 (8.70)	2 (4.35)	
Focal	2 (4.35)	4 (8.70)	
None	40 (86.96)	40 (86.96)	
White matter focal signal abnormality; *n* (%)			0.7486
Extensive	4 (8.70)	4 (8.70)	
Focal	9 (19.57)	11 (23.91)	
Linear	6 (13.04)	3 (6.52)	
None	27 (58.70)	28 (60.87)	
White matter myelination delay; *n* (%)			0.8404
Minimal	1 (2.17)	2 (4.35)	
Posterior limb of internal capsule (PLIC)	35 (76.09)	34 (73.91)	
PLIC and corona radiata	10 (21.74)	10 (21.74)	
Lateral ventricle diameter in mm; median (Q1–Q3)	5.51 (4.59–7.8625)	5.385 (4.4–6.905)	0.4701
Cortical gray matter signal abnormality; *n* (%)			0.6031
Extensive	1 (2.17)	1 (2.17)	
Focal	0 (0.00)	1 (2.17)	
None	45 (97.83)	44 (95.65)	
GM gyral maturation; *n* (%)			0.9999
Delay ≥ 4 weeks	3 (6.52)	3 (6.52)	
2 ≤ Delay < 4 weeks	14 (30.43)	14 (30.43)	
Delay < 2 weeks	29 (63.04)	29 (63.04)	
Deep gray matter signal abnormality; *n* (%)			0.9999
Extensive	1 (2.17)	1 (2.17)	
Focal	4 (8.70)	4 (8.70)	
None	41 (89.13)	41 (89.13)	
Deep gray matter volume reduction; median (Q1–Q3)	5.475 (4.74–5.962)	5.395 (4.695–5.935)	0.8759
Cerebellum signal abnormality; *n* (%)			0.6993
Extensive	2 (4.35)	2 (4.35)	
Focal	4 (8.70)	2 (4.35)	
None	40 (86.96)	42 (91.30)	
Cerebellum volume reduction; median (Q1–Q3)	2.33 (2.045–2.4975)	2.295 (2.145–2.5025)	0.9626
Intraventricular hemorrhage; *n* (%)			0.3970
No	25 (54.350)	29 (63.04)	
Yes	21 (45.65)	17 (36.96)	
Subdural hemorrhage; *n* (%)			0.3147
No	45 (97.83)	46 (100.00)	
Yes	1 (2.17)	0 (0.00)	

### ^1^H-MRS metabolite ratios in VP infants with different severity of brain damage

**Fig. 2:**
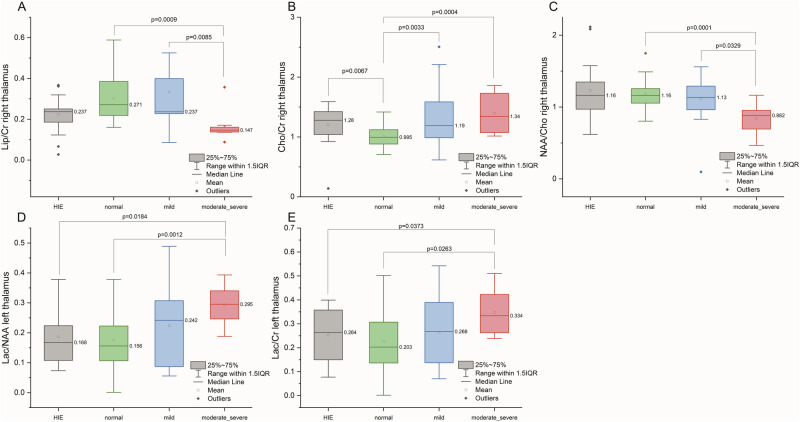
Baseline differences in ^1^H-MRS metabolite ratios among VP infants with various degrees of brain injury and infants with HIE after adjustment for postmenstrual age. **A**, **B**, **C** right thalamus, **D**, **E** left thalamus.

Compared with VP infants with a normal and with a mild GBAS, VP infants with a moderate/severe GBAS showed a lower NAA/Cho ratio in the right thalamus (*p* = 0.0001 and *p* = 0.0329, respectively) and a lower Lip/Cr ratio in the right thalamus (*p* = 0.0009 and *p* = 0.0085, respectively). Moreover, compared with VP infants with a normal GBAS, VP infants with a moderate/severe GBAS presented with a greater Cho/Cr ratio in the right thalamus (*p* = 0.0004) and greater Lac/NAA and Lac/Cr ratios in the left thalamus (*p* = 0.0012 and *p* = 0.0263, respectively) (Fig. 2, Table 4).

**Table 4:** Differences in ^1^H-MRS metabolite ratios among VP infants with various degrees of brain injury and infants with HIE before and after adjustment for postmenstrual age.

		HIE	p HIE vs. VP Unadjusted / adjusted for PMA	VP *n* = 46			*P* normal vs. mild Unadjusted / adjusted for PMA	*P* normal vs. moderate+severe Unadjusted / adjusted for PMA	*P* mild vs. moderate+severe Unadjusted / adjusted for PMA	*P* HIE vs. normal Unadjusted / adjusted for PMA	*P* HIE vs. mild Unadjusted / adjusted for PMA	*P* HIE vs. moderate+severe Unadjusted / adjusted for PMA
		*n* = 17		GBAS normal *n* = 23	GBAS mild *n* = 15	GBAS moderate+severe *n* = 8						
**Right thalamus TE 35** **ms**												
Lac/NAA	Median	0.1418	0.0549/	0.2193	0.1595	0.1899	0.8244/	0.3938/	0.4696/	0.0521/	0.3325/	0.0254/
	Q1	0.1010	0.4280	0.1185	0.0899	0.1560	0.8196	0.3919	0.4840	0.9311	0.7702	0.1378
	Q3	0.1865		0.2742	0.2269	0.3351						
Lip/Cr	Median	0.2370	0.0935/	0.2714	0.2367	0.1466	0.5763/	0.0007/	0.0074/	0.0243/	0.0481/	0.1353/
	Q1	0.1700	0.6358	0.2188	0.2201	0.1371	0.5823	0.0009	0.0085	0.7394	0.2527	0.0852
	Q3	0.2850		0.3863	0.4227	0.1660						
Lac/Cr	Median	0.1773	0.2424/	0.2373	0.1846	0.2288	0.5481/	0.3134/	0.7656/	0.4173	0.3282/	0.1956/
	Q1	0.1247	0.7843	0.1615	0.1367	0.1404	0.5515	0.3556	0.7783	0.4539	0.8156	0.3558
	Q3	0.2773		0.3263	0.3306	0.3412						
NAA/Cr	Median	1.3797	0.3355/	1.1303	1.2543	1.1655	0.0249/	0.5687/	0.0681/	0.0345	0.5908/	0.1043/
	Q1	1.1477	0.9870	1.0274	1.0148	0.8531	0.0243	0.4567	0.0639	0.2221	0.2818	0.1963
	Q3	1.6967		1.2711	1.8013	1.3695						
Cho/Cr	Median	1.2783	0.5261/0.6718	0.9945	1.1890	1.3428	0.0034/0.0033	0.0008/0.0004	0.4250/0.4169	0.0183/0.0067	0.1343/0.2404	0.1514/0.7716
	Q1	1.0384		0.8811	0.9860	1.0610						
	Q3	1.4410		1.1212	1.7415	1.7370						
NAA/Cho	Median	1.1618	0.1516/	1.1611	1.1333	0.8821	0.4528/	0.0002/	0.0604/	0.6206/	0.3796/	0.0039/
	Q1	0.9426	0.9848	1.0465	0.9336	0.6663	0.4562	0.0001	0.0329	0.4234	0.8349	0.0905
	Q3	1.4009		1.2706	1.3472	0.9552						
**Left thalamus TE 35** **ms**												
Lac/NAA	Median	0.1677	0.3919/	0.1561	0.2416	0.2953	0.1986/	0.0021/	0.1759/	0.7137/	0.3526/	0.0061/
	Q1	0.1023	0.5826	0.1068	0.0842	0.2393	0.2061	0.0012	0.1659	0.4978	0.4876	0.0184
	Q3	0.2408		0.2227	0.3111	0.3480						
Lip/Cr	Median	0.2741	0.2189/	0.2526	0.2832	0.2926	0.1603/	0.9135/	0.2998/	0.5592/	0.0777/	0.5655/
	Q1	0.1251	0.4837	0.1785	0.2303	0.1667	0.1606	0.7692	0.2902	0.8444	0.1335	0.7012
	Q3	0.3350		0.3332	0.3705	0.3647						
Lac/Cr	Median	0.2640	0.8454/	0.2027	0.2677	0.3338	0.4115/	0.0219/	0.1527/	0.4869/	0.7971/	0.0335/
	Q1	0.1427	0.8320	0.1365	0.1352	0.2590	0.4189	0.0263	0.1485	0.5960	0.8867	0.0373
	Q3	0.3636		0.3071	0.3921	0.4323						
NAA/Cr	Median	1.3868	0.1900/0.8847	1.2517	1.2177	1.1124	0.6417/0.6854	0.4563/0.3480	0.7106/0.5182	0.3933/0.9743	0.1486/0.6704	0.0860/0.3457
	Q1	1.2505		1.0776	1.0440	1.0438						
	Q3	1.5894		1.5021	1.3368	1.2623						
Cho/Cr	Median	1.0790	0.0981/	1.1705	1.3221	1.3190	0.3764/	0.4181/	0.9839/	0.3310/	0.0949/	0.0918/
	Q1	0.9745	0.2902	1.0590	1.1157	1.1525	0.3727	0.4162	0.9905	0.5939	0.2716	0.3193
	Q3	1.3138		1.4337	1.4437	1.6857						
NAA/Cho	Median	1.2248	0.0137/0.2734	0.9997	0.9554	0.8723	0.2100/0.2235	0.2202/0.1467	0.7685/0.5463	0.1188/0.6799	0.0018/0.0754	0.0079/0.1051
	Q1	1.0334		0.8621	0.8068	0.7141						
	Q3	1.3836		1.3369	1.0882	0.9356						

A, B, C right thalamus, D, E left thalamus.

### Comparison of ^1^H-MRS metabolite ratios between VP infants and full-term infants with HIE

There were no differences in the metabolite ratios of any thalamus between HIE and VP groups. Considering the differences between the HIE group and particular subgroups of VP infants, HIE group displayed lower Lac/NAA and Lac/Cr ratios in the left thalamus than did the moderate/severe brain damage group (Fig. 2).

## Discussion

Our study presents the brain metabolite analysis results of infants aged <32 gestational weeks who did or did not experience preterm brain injury. We subdivided our premature infant cohort according to the Kidokoro grading system and treated the obtained results as an outcome surrogate, as this scale has prognostic value for assessing brain damage in premature babies [9]. Moreover, we compared the obtained metabolite ratios with ratios calculated in term-born infants with HIE, who had the ^1^H-MRS performed according to the same technical protocol.

Interestingly, our study showed differences in metabolism among the left and right thalamus in the group of VP infants. On the one hand this observation indicates more uneven brain damage compared to HIE group. Conceivably the pathological processes that led to damage to the thalami in the VP group acted more locally, whereas in the HIE group they were expressed more globally. However, a comparison of imageable brain lesions seen on cranial ultrasound and/or MRI did not reveal significant differences between the two brain hemispheres. We speculate that subtle asymmetric brain injury may have resulted in the altered cellular metabolism that was observed exclusively by ^1^H-MRS. Our results indicate the limitations of using exclusively MRI to detect all pathologies that occurred in the brains of premature infants and signify a potential role for cerebral spectroscopy in adding more information to conventional brain MRI. Additionally, considering the potential consequences of uneven thalamic injury, they emphasize the need for long-term follow-up [10]. Prematurity may result in various negative effects on brain development, including decreased thalamic volume, altered thalamic shape, aberrant connectivity [11], and asymmetry in the thalami. Of note, Lao et al., in their shape and pose study of the thalami of the prematurely born infants, found a greater vulnerability of the left thalamus to prematurity associated changes [1]. Moreover, premature birth influences the development of brain lateralization [12]. Jayasundar et al., in their study on healthy adults, showed that metabolism of the corresponding areas in both brain hemispheres differed, which was an expression of brain lateralization [13]. It requires further research how the disruption of the development of brain lateralization influences thalamic metabolism in premature infants.

Our study showed that the thalamic metabolism differed among VP infants with different degrees of neonatal brain damage. We identified five metabolite ratios that differentiated the moderate/severe GBAS subgroup from the normal GBAS and mild GBAS subgroups: the NAA/Cho, Cho/Cr, Lip/Cr, Lac/NAA and Lac/Cr ratios. Additionally, the Lac/NAA and Lac/Cr ratios differed between infants in the moderate/severe GBAS subgroup and those in the HIE group.

In our study, the NAA/Cho ratio in the right thalamus in the moderate/severe GBAS subgroup was lower than that in the normal or mild GBAS subgroup, and a similar trend was shown in the left thalamus. NAA is a reliable noninvasive molecular imaging marker of functional neurons and its level decreases after hypoxia-ischemia [14]. The NAA/Cho ratio is used to assess neuronal integrity [15]. Notably, a decreased thalamic NAA/Cho ratio assessed in premature infants at TEA is considered a predictor of neurodevelopmental delay up to 24 months of age [16–18].

We assume, that an increased Cho/Cr ratio observed in VP infants with a higher degree of brain injury may result from a chronic inflammation, or other processes with heightened cell-membrane turnover, as demyelination, remyelination or gliosis, taking place at the site of examination [17].

Moreover, premature infants with the highest degree of brain damage were characterized by the lowest Lip/Cr ratio in the right thalamus. Lipids are normally visible in the ^1^H-MRS spectrum in the brain during the myelination process; therefore, we can suppose that the myelination process in the right thalamus in premature infants with moderate/severe brain damage is disturbed and not as active as that in premature infants with mild or no brain damage.

Lac is produced when brain aerobic glycolysis is reduced or ceased. In our study, in the left thalamus, the Lac/NAA and Lac/Cr ratios tended to increase with increasing severity of brain damage. When we compared normal GBAS subgroup with the moderate/severe GBAS subgroup, this difference reached statistical significance. Moreover, interestingly, the Lac/NAA ratio in the moderate/severe GBAS subgroup was even greater than that in the moderate/severe HIE group. In patients with HIE, high levels of Lac are observed on brain ^1^H-MRS, both in the acute phase and up to several months after a hypoxic-ischemic event in those with unfavorable neurological outcomes [19]. As Lac tends to accumulate in tissues affected by pathological processes such as necrosis, demyelination, cyst formation or inflammation [20], higher Lac/NAA and Lac/Cr ratios in the left thalamus of VP infants with moderate/severe brain injury may be associated with a greater degree of local brain damage, not only in the basal ganglia but also in adjacent tissues [21]. It is worth noting that such high Lac/NAA and Lac/Cr ratios were recorded at the age of expected delivery, many weeks after the initial intracranial event. The metabolic abnormalities observed in our premature infants may reflect brain metabolism in the chronic phase of neonatal brain injury. We suppose that preterm brain injury may also delay brain maturation and allow for longer persistence of higher lactate levels. Further observation of neurodevelopment in preterm infants is necessary, as the consequence of neonatal brain damage may change the trajectory of further brain development and delay brain maturation.

### Study strengths

We used the same scanner and technique to perform MRI + ^1^H-MRS in both study groups. Furthermore, data processing, analysis, and quantification were performed by the same medical physicist on a single device and in the same program. All these factors allowed us to compare the obtained results with a lower risk of error. Moreover, we assessed neonatal brain injury according to the Kidokoro scale, which is very detailed and has prognostic value.

### Limitations

In the HIE group, MRI + ^1^H-MRS was performed 7–14 days after birth. Preterm infants underwent these examinations at TEA. Therefore, it is possible that we examined different stages of evolution of primary brain damage. However, our findings suggest that brain damage, which occurred in preterm babies with a moderate/severe GBAS, may be even more devastating than that in babies with HIE; therefore, brain damage may have a vast impact on neurodevelopment in these children. Additionally, the number of HIE patients included to the study is relatively small. Moreover, we obtained different results for both thalami. However, there are studies that assessed only one thalamus, calculated the mean levels of thalamic metabolites, or obtained different results for both thalami as our study. The assessment of both thalami makes our work more precise, provides better insight into the metabolism of premature babies’ deep gray matter and may reveal local differences in metabolism depending on the degree of damage present at the cellular level.

## Conclusions

There were significant differences in the metabolite profiles measured at the time of expected delivery by ^1^H-MRS between VP infants with and without preterm brain injury.

Differences in the ^1^H-MRS metabolic ratios between premature infants with moderate/severe brain damage and hypothermia-treated infants with HIE may reflect profound metabolic alterations that persist for a long period of time in preterm patients.

^1^H-MRS examination indicates a loss of integrity of neurons, a disturbed myelination and a long-term persistence of an inflammatory state as possible pathomechanisms present in VP infants with moderate/severe preterm brain injury.

Observed spectroscopic differences between both thalami may suggest that local injuries at a cellular level are not visible on conventional MRI but affect the metabolism of specific brain regions.

## Supplementary information

Supplementary material

## Data Availability

All the data analysed during this study are included in this article. Further inquiries can be directed to the corresponding author.

## References

[1] Lao Y, Wang Y, Shi J, Ceschin R, Nelson MD, Panigrahy A, et al. Thalamic alterations in preterm neonates and their relation to ventral striatum disturbances revealed by a combined shape and pose analysis. Brain Struct Funct. 2016;221:487–506. 10.1007/s00429-014-0921-7. 25366970 10.1007/s00429-014-0921-7 PMC4417103

[2] Kidokoro H, Neil JJ, Inder TE. New MR imaging assessment tool to define brain abnormalities in very preterm infants at term. AJNR Am J Neuroradiol. 2013;34:2208–14. 10.3174/ajnr.A3521. 23620070 10.3174/ajnr.A3521 PMC4163698

[3] Lally PJ, Montaldo P, Oliveira V, Soe A, Swamy R, Bassett P, et al. Magnetic resonance spectroscopy assessment of brain injury after moderate hypothermia in neonatal encephalopathy: a prospective multicentre cohort study. Lancet Neurol. 2019;18:35–45. 10.1016/S1474-4422(18)30325-9. 30447969 10.1016/S1474-4422(18)30325-9 PMC6291458

[4] Blüml S, Wisnowski JL, Nelson MD Jr, Paquette L, Panigrahy A. Metabolic maturation of white matter is altered in preterm infants. PLoS ONE. 2014;9:e85829 10.1371/journal.pone.0085829. 24465731 10.1371/journal.pone.0085829 PMC3899075

[5] Xu D, Vigneron D. Magnetic resonance spectroscopy imaging of the newborn brain-a technical review. Semin Perinatol. 2010;34:20–7. 10.1053/j.semperi.2009.10.003. 20109969 10.1053/j.semperi.2009.10.003 PMC2842012

[6] Thayyil S, Chandrasekaran M, Taylor A, Bainbridge A, Cady EB, Chong WK, et al. Cerebral magnetic resonance biomarkers in neonatal encephalopathy: a meta-analysis. Pediatrics. 2010;125:e382–95. 10.1542/peds.2009-1046. 20083516 10.1542/peds.2009-1046

[7] Committee on Fetus and Newborn, Papile LA, Baley JE, Benitz W, Cummings J, Carlo WA, et al. Hypothermia and neonatal encephalopathy. Pediatrics. 2014;133:1146–50. 10.1542/peds.2014-0899. 24864176 10.1542/peds.2014-0899

[8] Weeke LC, Groenendaal F, Mudigonda K, Blennow M, Lequin MH, Meiners LC, et al. A novel magnetic resonance imaging score predicts neurodevelopmental outcome after perinatal asphyxia and therapeutic hypothermia. J Pediatr. 2018;192:33–40.e2. 10.1016/j.jpeds.2017.09.043. 29246356 10.1016/j.jpeds.2017.09.043 PMC5743051

[9] Jansen L, van Steenis A, van den Berg-Huysmans AA, Wiggers-de Bruine ST, Rijken M, de Vries LS, et al. Associations between neonatal magnetic resonance imaging and short- and long-term neurodevelopmental outcomes in a longitudinal cohort of very preterm children. J Pediatr. 2021;234:46–53.e2. 10.1016/j.jpeds.2021.02.005. 33577803 10.1016/j.jpeds.2021.02.005

[10] De Jong LW, van der Hiele K, Veer IM, Houwing JJ, Westendorp RG, Bollen EL, et al. Strongly reduced volumes of putamen and thalamus in Alzheimer’s disease: an MRI study. Brain. 2008;131:3277–85. 10.1093/brain/awn278. 19022861 10.1093/brain/awn278 PMC2639208

[11] Ball G, Boardman JP, Rueckert D, Aljabar P, Arichi T, Merchant N, et al. The effect of preterm birth on thalamic and cortical development. Cereb Cortex. 2012;22:1016–24. 10.1093/cercor/bhr176. 21772018 10.1093/cercor/bhr176 PMC3328341

[12] Scheinost D, Lacadie C, Vohr BR, Schneider KC, Papademetris X, Constable RT, et al. Cerebral lateralization is protective in the very prematurely born. Cereb Cortex. 2015;25:1858–66. 10.1093/cercor/bht430. 24451659 10.1093/cercor/bht430 PMC4459290

[13] Jayasundar R, Raghunathan P. Evidence for left-right asymmetries in the proton MRS of brain in normal volunteers. Magn Reson Imaging. 1997;15:223–34. 10.1016/s0730-725x(96)00342-6. 9106150 10.1016/s0730-725x(96)00342-6

[14] Shibasaki J, Niwa T, Piedvache A, Tomiyasu M, Morisaki N, Fujii Y, et al. Comparison of predictive values of magnetic resonance biomarkers based on scan timing in neonatal encephalopathy following therapeutic hypothermia. J Pediatr. 2021;239:101–109.e4. 10.1016/j.jpeds.2021.08.011. 34391766 10.1016/j.jpeds.2021.08.011

[15] Peden CJ, Cowan FM, Bryant DJ, Sargentoni J, Cox IJ, Menon DK, et al. Proton MR spectroscopy of the brain in infants. J Comput Assist Tomogr. 1990;14:886–94. 10.1097/00004728-199011000-00004. 2229562 10.1097/00004728-199011000-00004

[16] Hyodo R, Sato Y, Ito M, Sugiyama Y, Ogawa C, Kawai H, et al. Magnetic resonance spectroscopy in preterm infants: association with neurodevelopmental outcomes. Arch Dis Child Fetal Neonatal Ed. 2018;103:F238–F244. 10.1136/archdischild-2016-311403. 28724545 10.1136/archdischild-2016-311403

[17] Cebeci B, Alderliesten T, Wijnen JP, van der Aa NE, Benders MJNL, de Vries LS, et al. Brain proton magnetic resonance spectroscopy and neurodevelopment after preterm birth: a systematic review. Pediatr Res. 2022;91:1322–33. 10.1038/s41390-021-01539-x. 33953356 10.1038/s41390-021-01539-x

[18] Laccetta G, De Nardo MC, Cellitti R, Angeloni U, Terrin G. ^1^H-magnetic resonance spectroscopy and its role in predicting neurodevelopmental impairment in preterm neonates: a systematic review. Neuroradiol J. 2022;35:667–77. 10.1177/19714009221102454. 35698266 10.1177/19714009221102454 PMC9626842

[19] Robertson NJ, Cox IJ, Cowan FM, Counsell SJ, Azzopardi D, Edwards AD. Cerebral intracellular lactic alkalosis persisting months after neonatal encephalopathy measured by magnetic resonance spectroscopy. Pediatr Res. 1999;46:287–96. 10.1203/00006450-199909000-00007. 10473043 10.1203/00006450-199909000-00007

[20] Liserre R, Pinelli L, Gasparotti R. MR spectroscopy in pediatric neuroradiology. Transl Pediatr. 2021;10:1169–1200. 10.21037/tp-20-445. 34012861 10.21037/tp-20-445 PMC8107850

[21] Montaldo P, Ivain P, Lally P, Bassett P, Pant S, Oliveira V, et al. White matter injury after neonatal encephalopathy is associated with thalamic metabolite perturbations. EBioMedicine. 2020;52:102663 10.1016/j.ebiom.2020.102663. 32062359 10.1016/j.ebiom.2020.102663 PMC7016374

