# Supplementary material for: Cerebral magnetic resonance spectroscopy – insights into preterm brain injury
Source: J Perinatol. 2024 Nov 28;45(2):194–201. doi: 10.1038/s41372-024-02172-2 (PMC11825355; doi:10.1038/s41372-024-02172-2)
Supplement: Supplementary file 1 — Supplementary material [file 41372_2024_2172_MOESM1_ESM.docx]

**MRI and ^1^H-MRS acquisition**

Before MRI and ^1^H-MRS examinations, a neonatologist assessed all the study participants to ensure that they were clinically stable enough to undergo scanning. Physiologic parameters (heart rate, oxygen saturation) were monitored with a visual display in the MRI control room. Each infant was provided with thermal comfort through special covers. Dedicated earmuffs were used for hearing protection. All the examinations were supervised by an anesthesiologist and NICU nurse experienced in MRI procedures. Patients were treated with feed and swaddle technique. In the case of a prolonged examination, a single dose of sedative was allowed according to the protocol. The administration of sedative medication was the decision of the anesthesiologist.

MRI and ^1^H-MRS were performed using the 1,5 T GE Optima 450w whole-body magnetic resonance scanner with a 20-channel head coil. The MR system was equipped with strong whole-body gradients ensuring an amplitude of 45 mT/m and a rise rate of 120 T/m/s on each axis, thus providing fast, accurate and highly repeatable scans. MRI and ^1^H-MRS examinations were performed on the same magnetic resonance scanner and on the same day. The total measurement time was approximately 50 min.

In the first step, each infant underwent non-contrast structural brain imaging.

The non-contrast brain imaging protocol included the following sequences:

1. T2 sequence in axial plane (scanning sequences: fast spin echo; slice thickness 4,0 mm; spacing 2,0 mm; TR 6000 ms; TE 97 ms; FOV 24 cm and matrix 320x320)
2. T2 sequence in sagittal plane (scanning sequences: fast spin echo; slice thickness 4,0 mm, spacing 2,0 mm TR 3660 ms, TE 88 ms, FOV 24 cm, matrix 384x224)
3. T2 sequence in coronal plane (scanning sequences: fast spin echo; slice thickness 4,0 mm, spacing 2,0 mm TR 4600 ms, TE 88 ms, FOV 24 cm, matrix 384x224)
4. T2 FLAIR in axial plane (scanning sequences: spin echo inversion recovery; slice thickness 4,0 mm, spacing 2,0 mm, TR 8000 ms, TE 123 ms, T1 8000 ms, FOV 24 cm, matrix 288x288)
5. T1 sequence in axial plane (scanning sequences: spin echo slice thickness 4,0 mm, spacing 2,0 mm, TR 320 ms, TE 9 ms, FOV 24 cm, matrix 512x224)
6. GRE T2 sequence in axial plane (scanning sequences: gradient echo slice thickness 4,0 mm, spacing 2,0 mm, TR 720 ms, TE 15 ms, flip angle 20, FOV 24 cm, matrix 320x192)
7. 3D T1 sequence in axial, coronal and sagittal plane (scanning sequences: gradient echo inversion recovery; slice thickness 2,0 mm, spacing -1,0 mm, TR 10 ms, TE 4,4 ms, TI 450 ms, flip angle 12, FOV 20 cm, matrix 320x192).

Additionally, the following sequences were made: diffusion-weighted imaging (DWI), diffusion tensor imaging (DTI) and arterial spin labeling (ASL). DWI was performed using parameter b values of 0 and 1000 s/mm2. The apparent diffusion maps were automatically calculated. DTI for b values 0 and 1000 s/mm2 was oriented in 128 directions. The colored orientation, fractional anisotropy, average diffusion coefficient, isotropic image, volume ratio anisotropy, exponential attenuation and T2-weighted trace maps were automatically calculated. Non-contrast perfusion (ASL) was performed using EPI sequence with 1250 ms delay. ASL included inline calculation of cerebral blood flow (relCBF) maps for a quantitative evaluation of perfusion.

The next step was ^1^H-MRS, which was performed using single-voxel spectroscopy (SVS) and multivoxel techniques (2D CSI – chemical shift imaging). MRS examination was based on the PRESS technique (Point-Resolved Spectroscopy Sequence). The PRESS sequence utilizes a 90^0^ and two 180^0^ radiofrequency pulse. The CHESS sequence (CHEmical shift Selective Imaging Sequence) was implemented for water suppression with a frequency-selective 90° pulse and dephasing gradient to destroy the water signal. The acquisition parameters of ^1^H-MRS were TE 35 ms and 144 ms, TR 2000 ms, 64 averages acquired. Two echo times (TE) were selected to record metabolites with both long and short relaxation times. The local magnetic field homogeneity was optimized by auto-shim procedure. The quality of the shimming obtained in the voxel was controlled by the spectral line width (full width of half maximum (FWHM) in Hz) of the unsuppressed water, obtained by the automated optimization sequence before scanning. The FWHM and SNR (signal to noise ratio) values ​​were used to exclude low quality MR spectra. The spectra for FWHM < 10 MHz and SNR above 90 were analyzed.

Two-dimensional T2 imaging was performed to visualize anatomical brain structures and planning volume of interest (VOI) position for spectroscopy. The VOI size was adjusted to the anatomical size of the thalamus and was approximately 1.69 cm^3^.

^1^H-MRS data were analyzed using SAGE software (Spectroscopy Analysis by GE) [1] and on AW (Advantage Workstation, GE) 4.5 workstation with Functool. Postprocessing steps included:

- loading of appropriate raw data presented in the form of FID signal;
- data reconstruction (reconstruct-Probe Quant), which consists of noise reduction by applying filters and automatic Fourier transformation (conversion of a time-domain function to a frequency-domain function);
- offset correction-subtraction of the signal component that arose in the receiver because of the interaction of electronic circuits, while the FID signal disappeared to zero;
- zero filling-supplementing the digital form of the signal with additional data of zero amplitude to improve the resolution of the spectrum;
- apodization-signal multiplication by appropriate mathematical functions, which improves the signal-to-noise ratio;
- determination of peaks that were subject to further analysis;
- converting the spectrum with marked peaks into the FID signal (processing FID generate);
- Fourier transformation of the newly received FID signal;
- reading the value of the area of the fields under the selected peaks;
- superimposing the developed spectrum with the selected peaks on the original spectrum.

The following metabolites were manually selected from the spectrum: lipids (Lip) (0.9-1.0 ppm), lactates (Lac) (1.33 ppm), alanine (Ala) (1.48 ppm), N-acetyl aspartate (NAA) (2.02 ppm), γ-amino butyric acid (GABA) (2.3 ppm), glutamine and/or glutamate (GLX2) (2.45 ppm), glutamine (Gln) (2.9 ppm), creatine (Cr) (3.02 ppm), choline (Cho) (3.22 ppm), glucose (GLC1) (3,43 ppm), myo-Inositol (mI) (3.56 ppm), glutamate (GLX3) (3.6-3.8 ppm), and glucose (GLC2) (3.8 ppm).

Once peaks were selected, SAGE software automatically calculated the peak area that correlates with metabolite concentration in the VOI. MRI of the brain, ^1^H-MRS and spectrum analysis were performed by a radiologist and medical physicist with over 10 years of experience. Metabolite concentrations were compared for the same test parameters, i.e., the same TE. A TE of 35 ms has been selected. We focused our analyses on five key metabolites in the bilateral thalamus (NAA, Cho, Cr, Lip, Lac) due to the proven linking of changes in their ratios with brain pathologies in premature newborns. The ratios of individual metabolite concentrations to Cr, Cho and NAA were calculated.

References:

1. Shih CM, Lai JJ, Chang CC, Chen CS, Yeh YC, Jaw TS, et al. Comparison of LCModel and SAGE in Analysis of Brain Metabolite Concentrations-A study of Patients with Mild Cognitive Impairment. Acta Neurol Taiwan. 2017 Mar 15;26(1):20-28. PMID: 28752510
